# Biodegradable Nasal Packings for Endoscopic Sinonasal Surgery: A Systematic Review and Meta-Analysis

**DOI:** 10.1371/journal.pone.0115458

**Published:** 2014-12-19

**Authors:** Maoxiao Yan, Dandan Zheng, Ying Li, Qiaoli Zheng, Jia Chen, Beibei Yang

**Affiliations:** 1 Department of Otorhinolaryngology, The Second Affiliated Hospital, School of Medicine, Zhejiang University, Hangzhou, China; 2 Clinical Research Center, The Second Affiliated Hospital, School of Medicine, Zhejiang University, Hangzhou, China; Beijing Institiute of Otolaryngology, China

## Abstract

**Objectives:**

To assess biodegradable nasal packing effectiveness for improving postoperative symptoms and mucosal healing after endoscopic sinonasal surgery as compared with conventional/non-packing groups.

**Methods:**

Relevant articles were searched on PubMed, EMBASE, and the Cochrane Central Register of Controlled Trials. We included randomized controlled trials (RCTs) that compared biodegradable packings with conventional packings or no packing, reporting postoperative symptoms and/or mucosal healing outcomes.

**Results:**

This review included 19 studies, of which 11 compared biodegradable packings with conventional packings. Meta-analysis found that biodegradable packings significantly improved postoperative symptoms: bleeding at removal, pain at removal, pain *in situ*, and nasal blockage. Mucosal healing outcomes were inconsistent within studies, with no data could be pooled. Eight studies compared biodegradable packings with non-packing group. Postoperative symptom data in this comparison could not be pooled: A protective or equal effect on postoperative bleeding was reported in different studies; no difference was reported in pain status and nasal blockage. As for mucosal healing, meta-analysis showed that two arms of comparison had similar effect on synechiae, edema, infection and granulation at each time point.

**Conclusion:**

The limiting evidence suggests that biodegradable nasal packings are statistically better than conventional packings in postoperative symptoms, and probably comparable to non-packing group, as in this comparison we could not carry out meta-analysis. No beneficial or detrimental effect on postoperative mucosal healing could be determined based on existing evidence.

## Introduction

### Background

Chronic rhinosinusitis (CRS), septal deviation, and inferior turbinate hypertrophy are among the most common diseases seen in the ENT department; these affect patients of all ages and both genders. They can cause recurrent or persistent nasal obstruction and/or a runny nose. Sometimes, they may induce anosmia, headache, dizziness, and/or insomnia, thus having a significant impact on life quality. Operations, such as endoscopic sinus surgery (ESS), septoplasty and conchotomy, are often unavoidable when medical treatments have failed. Traditionally, at the close of operations, nasal packings are placed into the nasal cavities to prevent bleeding of the wound and provide a “scaffold” for wound healings. These years, the development of functional endoscopic sinus surgery (FESS) and mucosal-sparing technique has made postoperative bleeding less threatening [1]. Therefore, the nasal packing status has been challenged and re-discussed over the years. Rhinologists nowadays highly value minimal complications, satisfying life quality and optimized mucosal healing when choosing a postoperative treatment regimen [2].

Conventional nasal packings include those common-used removable materials like gauze, cotton, and sponge, whether they are coated by glove fingers or any chemicals. Merocel, made from inflatable polyvinyl acetate sponge, is a typical conventional removable nasal packing. These packings have several advantages include cheap price, easy manipulation, and sufficient supporting ability.

However, conventional packings are criticized for their multiple defects. These include nasal airway obstruction, headache/pressure, and painful mouth and pharynx dryness due to prolonged oral breathing. Prolonged packing time may incur infection. Removal of the packing usually causes tremendous discomfort - some patients consider it the most objectionable part of the whole procedure [3]. Additionally, packing removal can cause extra mucosal disturbances resulting in bleeding.

These drawbacks associated with removable nasal packings have led to ongoing development of biodegradable/absorbable biomaterials not requiring subsequent removal. The followings products are biodegradable or absorbable nasal packings.

Synthetic Polyurethane foam (NasoPore) is one of the most common absorbable products used for nasal surgery. The polyurethane bonds provide strong initial compressive mechanical properties, while the hydrophilic component facilitates water uptake and rapid fragmentation [4].

Cutanplast is a hemostatic gelatin sponge product made from 99.7% pig gelatin, and is absorbable, water-insoluble, and digestible by trypsin [5]. The porous surface of gelatin induces rapid blood plaque rupture with the consequent activation of coagulation cascade [6]. Gelfoam is also an absorbable gelatin sponge with different gelatin density and porosity, which is widely used in ear and brain-related procedures [7]. FloSeal is a paste of bovine gelatin particles combined with thrombin; it can be injected into the dissected ethmoid cavity [8].

Hyaluronic acid, a linear polysaccharide and naturally occurring extracellular matrix constituent, is designed in bioresorbable nasal packings (MeroGel and MeroPack). Hyaluronic acid keeps the surgical site moist, reduces adhesions, and decreases healing time [9].

Carboxymethylcellulose (CMC) is a vegetable-based polysaccharide foam that actively promotes platelet aggregation upon blood contact. Stammberger Sinu-Foam is made of dissolvable CMC foam. Starting as a dry CMC fiber within a syringe, the CMC forms a viscous gel when mixed properly with sterile water [10]. It easily conforms to the nasal and sinus cavities during placement; it provides a moist, hydrocolloid physical barrier naturally dissolving over several days. Both CMC mesh (Rapid Rhino Sinu-Knit) and CMC gels (Rapid Rhino Sinus dressing) are CMC foam products.

Fibrin glue (Quixil) is a surgical sealant whose formulation is based on human clottable proteins (virus-inactivated cryo concentrate) and a highly-purified native human thrombin. This fibrin glue attaches firmly to tissue, thereby achieving instant hemostasis. Fibrin glue is a biological product of human origin, naturally metabolized within several days without causing inflammation and crusts [11].

Microporous polysaccharide hemisphere (MPH) powder is another absorbable hemostatic agent. It is a rapidly-cleared powder of microporous particles produced from purified potato starch, acting as a molecular sieve to extract fluids from blood. This causes the particles to swell and concentrate serum proteins, platelets, and other formed elements on their surfaces. The spherical particles and their coating of cellular elements create a scaffold for robust clot formation [12].

Chitosan, prepared from chitin, has been long known to be an effective hemostatic agent [13]. A novel gel has been formed by cross-linking chitosan and dextran derivatives (CD gel) for use as a hemostatic agent after nasal surgery [14].

The word “absorbable” is often phrased in existing articles relative to new packing products. Materials such as CMC are not absorbable, but can be spontaneously degraded/dissolved and then either washed by nasal irrigation or sucked by a suction device [10]. Thus, we used the term “biodegradable” to summarize all included biomaterials.

These promising products are expected to attain better hemostasis, greater comfort, less synechiae formulation, less infection and improved mucosal healing. However, clinical researches have reported inconsistent results. As the biodegradable materials often are expensive, the cost-effectiveness of the material remains controversial. Meanwhile, with the development of surgical technique, a rising number of surgeons are advocating the conception of “no packing after ESS” which is also under heated discussion.

### Objectives

We performed this meta-analysis to assess the effectiveness of biodegradable nasal packings in improving postoperative symptoms and wound healing after sinonasal surgery. The packings were compared to either conventional packings or non-packing group.

## Methods

### Search Strategy, Inclusion and Exclusion Criteria

A computer search of the literature was conducted, including PubMed, EMBASE and the Cochrane Central Register of Controlled Trials (CENTRAL) up to April, 2014. The following search terms were used: (pack* OR sponge OR gauze OR gelatin OR foam OR “polyvinyl acetate” OR Merocel OR NasoPore OR carboxymethylcellulose OR hyaluron* OR chitosan OR “fibrin glue”) AND (nose OR nasal OR sinonasal OR paranasal OR endonasal OR septal* OR septum OR sinus* OR rhinosinusitis OR nasosinusitis OR pansinusitis), filtered by species (human) and language (English). We reviewed the reference lists from retrieved articles and reviews in order to identify further relevant studies.

Studies were eligible for inclusion if they met the following criteria:

1. Research design: RCTs
2. Language: English
3. Participants: patients who underwent any type of sinonasal surgeries as below:
  - FESS or termed as endoscopic sinus surgery (ESS) for chronic rhinosinusitis (CRS)
  - Septoplasty for deviated nasal septum
  - Conchotomy or turbinectomy for hypertrophy of the inferior turbinate
4. Interventions: at the conclusion of surgery, nasal cavities randomized to the experiment group were managed with biodegradable packings; the remaining patients were in a control group comprised of conventional packings or no packing. Other routine adjuvant postoperative therapies such as nasal saline spray, intranasal corticosteroids, and antibiotics should be equivalent in the two groups.
5. Outcomes: studies should contain data for at least one of the following:
  - Symptoms associated with nasal packing: bleeding with *in situ* packing, bleeding at removal, pain *in situ*, and nasal blockage.
  - Recovery of nasal mucosa: mucosal edema, synechiae, infection and granulation.

Studies were excluded if:

1. Participants underwent other surgeries beyond the three previously-mentioned types, such as resection of fibroangioma, carcinoma, or other related procedures.
2. Nasal cavities received more than one kind of packings, or a non-biodegradable material coated with biodegradable material, such as Rapid Rhino Riemann [15], Visco [16], and so on.

### Data extraction and quality assessment

The following information was retrieved independently by two authors from each publication: first author, publication year, participant characteristics (type of surgery before packing, age, number of enrolled patients, and attrition), interventions (packing methods), and previously-mentioned outcomes. Inconsistencies in research were resolved through debate and consultations. If any information was not mentioned in the original study, the item would be classified as “Not Available (NA)”. The extracted data were entered using Microsoft Excel 2010 and RevMan 5.2 [17] and were checked by a third author. The quality of studies was examined using a Cochrane Collaboration tool for assessing the risk of bias. The standard “Risk of bias” table includes assessments for sequence generation, allocation sequence concealment, blinding of participants and personnel, outcome assessment blinding, incomplete outcome data, selective outcome reporting, and “other issues”. The risk of each bias was judged as “low”, “high” or “unclear” as described in *The Cochrane Handbook for Systematic Reviews of Interventions* (*The Cochrane Handbook*) [18].

### Statistical Analysis

Relative risks (RRs) and related 95% confidence intervals (CIs) of pooled results were estimated for dichotomous outcomes. Standardized mean differences (SMDs) and 95% CIs were estimated for continuous data. Ordinal outcomes were summarized using dichotomous data methods (see *The Cochrane Handbook*) [18]. Heterogeneity was tested by using the I^2^ statistic, and studies were considered to have low (I^2^ = 25–49%), moderate (I^2^ = 50–74%) or high (I^2^>75%) heterogeneity [19]. The random-effect model was used when I^2^>50%, whereas the fixed-effect model was used in cases where heterogeneity was not significant (I^2^<50%). Whenever heterogeneity was present, we performed sensitivity analyses in order to investigate the single study influence on the overall result. This was done by excluding one study per analysis. Subgroup analysis was performed where necessary. As the number of studies for each comparison was less than ten, no funnel plot was created to access the likelihood of publication bias [19]. All the statistical analyses were performed using Review Manager (RevMan) 5.2 [17].

## Results

### Study selection and characteristics

**Figure 1:**
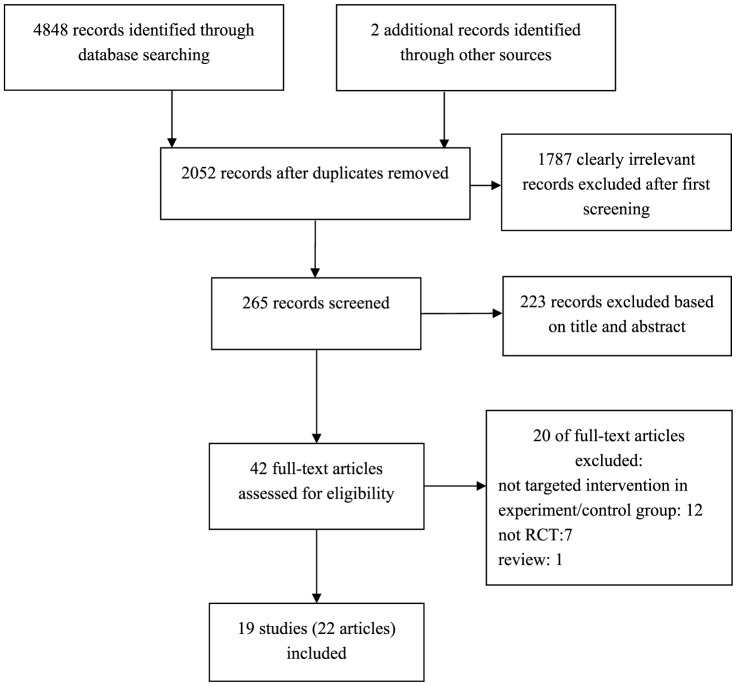
Flow diagram of article selection for inclusion.

A total of 4850 references were identified from the searches (2022 from PubMed, 2250 from Embase, 576 from the Cochrane library; and 2 from included article references): 4585 of these were removed because of duplicate and clearly irrelevant references, 223 of the remainder were excluded due to title and abstract, leaving 42 full-texts for further consideration. A flow chart of study retrieval and selection is provided in Fig. 1.

The final analysis included 19 studies (22 articles) published between 2003 and 2013. Three reports by Vaiman *et al*. published in 2002 and 2005 were from a single study [11], [20], [21], the two by Antisdel *et al*. in 2011 and 2009 were from one study as well [12], [22]. Eleven studies compared biodegradable nasal packing with standard non-biodegradable packing, and eight were compared with no packing. All studies recruited adult participants except one study by Hu *et al*. that focused on pediatric patients [23]. Some studies used intra-patient controls: each patient had both nasal cavities that received different interventions; every patient provided two samples. In other studies, each patient was regarded as one sample, as each patient received only one kind of intervention. Therefore, we enrolled a total of 2063 samples from 1403 patients. The characteristics of all included studies in the analysis are summarized in Table 1.

**Table 1:** Characteristics of included studies.

Author & publish year	Sample/patient size	Attrition	Age range/mean (years)	Surgery	Intervention	Outcome	
						Postoperative Symptom	Mucosal healing
**Andreas 2009**	80 samples/40 patients	0.0%	NA/49.9	ESS	CMC VS. no packing	Nasal blockage	NA
**Antisdel 2011 (2 articles)**	80 samples/40 patients	0.0%	NA/48.2	ESS	MPH VS. no packing	Postoperative Bleeding	Synechiae, edema, infection
**Berlucchi 2009**	88 samples/66 patients	14.0%	NA	ESS	MeroGel VS. conventional packing	NA	Synechiae, edema, granulation tissue
**Cho 2012**	210 samples/105 patients	4.7%	20–76/35.7	ESS	Cutanplast VS. conventional packing	Pain at removal, bleeding at removal	Postoperative wound healing, Lund-Kennedy scores
**Franklin 2007**	140 samples/70 patients	0.0%	18–80/NA	ESS	MeroGel VS. conventional packing	Symptom score questionnaire	Endoscopic severity score
**Hu 2008**	120 samples/60 patients	0–10.0%	7–15/10.6	ESS	Meropack VS. no packing	postoperative bleeding	Synechiae, infection, granulation tissue
**Kastl 2008**	82 samples/41 patients	2.4–14.6%	NA/49.8	ESS	CMC VS. no packing	Postoperative bleeding	NA
**Kastl 2009**	54 samples/54 patients	3.7%	NA/50.3	ESS	CMC VS. no packing	NA	Synechiae, granulation tissue, infection
**Kim 2013**	70 samples/70 patients	25.7%	NA/20.32	Conchotomy	NasoPore VS. conventional packing	Bleeding at/after removal, pain at removal	NA
**kim yoo 2011**	60 samples/60 patients	0.0%	19–64/40.2	Septoplasty	NasoPore VS. conventional packing	Bleeding at removal, pain in situ, pain at removal	NA
**Miller 2003**	74 samples/37 patients	0–24.3%	NA/39.1	ESS	MeroGel VS. conventional packing	NA	Synechiae, edema, infection
**Shoman 2009**	60 samples/30 patients	0.0%	29–76/54	ESS	NasoPore VS. conventional packing	Bleeding at removal, pain in situ, pain at removal, nasal blockage	NA
**Szczygielski 2010**	60 samples/60 patients	10.0%	19–71/43.2	ESS	CMC VS. conventional packing	Postoperative bleeding, pain in situ	Synechiae
**Valentine 2010**	80 samples/40 patients	0–10.0%	20–80/49.5	ESS	CD gel VS. no packing	Nasal blockage, pain in situ	Synechiae, edema, infection, granulation tissue
**Vaiman 2005 (3 articles)**	513 samples/513 patients	3.7%	NA/34.4	ESS, Septoplasty, conchotomy	Quixil VS. conventional packing	Postoperative bleeding	NA
**Verim 2013**	116 samples/58 patients	3.4%	17–67/41.6	ESS	NasoPore VS. conventional packing	Bleeding at removal, pain at removal, nasal blockage	Lund-Kennedy endoscopic scoring
**wee 2012**	42 samples/21 patients	0.0%	12–75/39.7	FESS with or without septoplasty	Gelfoam VS. no packing	Nasal blockage, postoperative bleeding	Synechiae, granulation tissue, edema
**Wormald 2006**	84 samples/42 patients	4.8–9.5%	NA/41.5	ESS	Merogel VS. no packing	NA	Synechiae, edema, infection, Lund-MacKay Scores
**Yilmaz 2013**	50 samples/50 patients	8.0%	NA/29.5	Septoplasty	NasoPore VS. conventional packing	Bleeding at removal, pain in situ, pain at removal, nasal blockage	NA

ESS, endoscopic sinus surgery; NA, not available; CMC, Carboxymethylcellulose; MPH, Microporous polysaccharide hemispheres; CD gel, chitosan and dextran gel.

### Risk of bias in included studies

**Figure 2:**
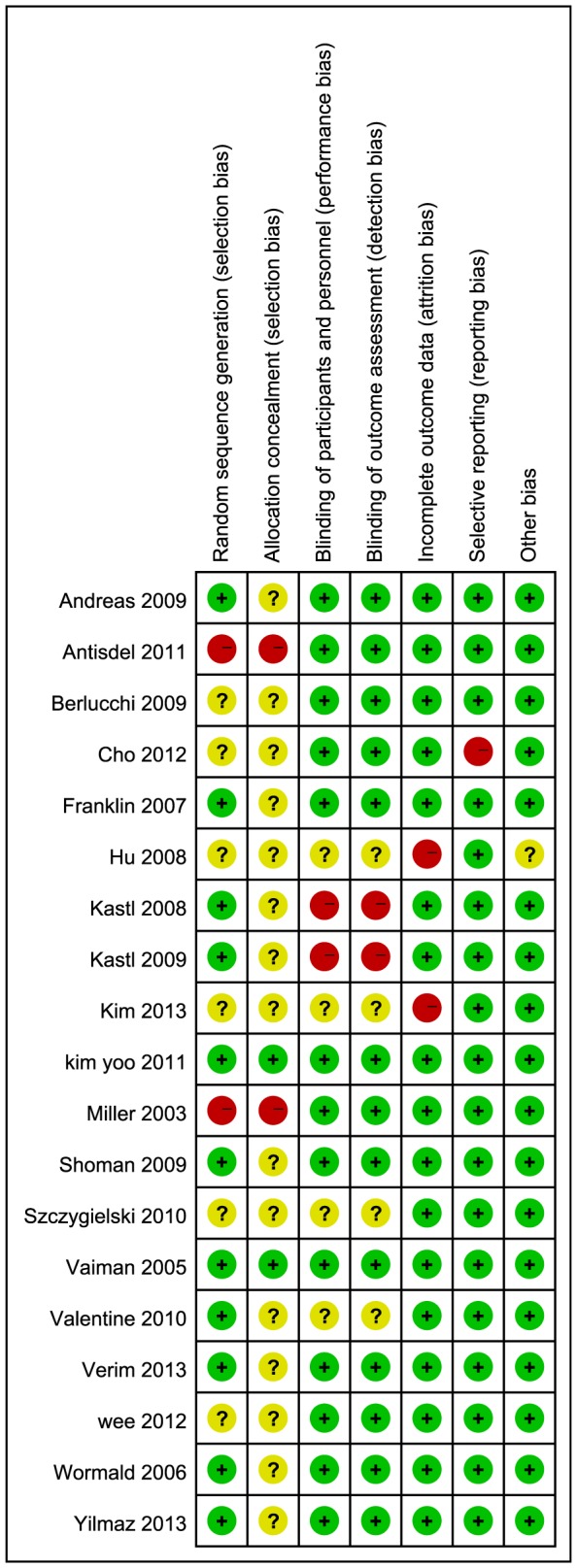
Risk of bias summary: review authors' judgments about each risk of bias item for each included study.

The overall methodological study quality was assessed by the Cochrane Collaboration tool for the risk of bias (Fig. 2, **Risk of bias summary**). Since nasal packing was a type of surgical procedure, and differences in packing material characteristics were often obvious, it was hard to blind the surgeons during research. Outcomes were not likely to be influenced by lack of blinding during the packing procedure, if blinding was well-performed during outcome measurement. Therefore, we judged the risk of performance bias risk similar to detection bias (see Table 8.5.d, **Criteria for judging risk of bias**, *The Cochrane Handbook* [18]).

Generally, included studies had a low risk of bias for selective reporting and incomplete outcome data, a medium risk of bias for blinding in performance/detection and random sequence generation, and a high risk of bias for allocation concealment. With regard to “other bias”, one study by Hu *et al*. that focused on children gave little information related to randomization and blinding [23]. We judged that study to have an “unclear risk” of other bias as insufficient information to assess the existing risk of bias.

### Effects of interventions

In septoplasty, nasal packing prevents postoperative bleeding and septal hematoma, [24]. In conchotomy, packing is used to control bleeding, too [25]. Particularly, besides its hemostatic effect, nasal packing in ESS also plays a role in mucosal recovery. Hence, all studies were assigned to postoperative symptom evaluation, while only those concerning ESS were included for an investigation of mucosal healing.

### 1. Biodegradable packings vs. conventional packings

**Figure 3:**
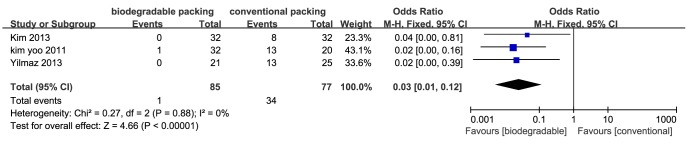
Forest plot of comparison: bleeding at removal (biodegradable packings versus conventional packings) - for dichotomous data.

**Figure 4:**
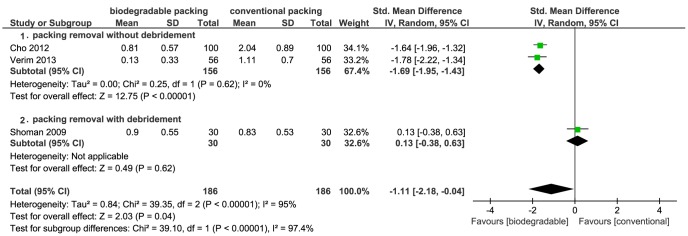
Forest plot of comparison: bleeding at removal (biodegradable packings versus conventional packings) - for continuous data.

- Symptoms associated with packing

1. **Bleeding in situ and septal hematoma.** Preventing postoperative bleeding is the primary function of nasal packing. Most of the relevant studies did not present numeric data about postoperative bleeding in this comparison, except Szczygielski et al.'s study reporting dichotomous data and Shoman et al.'s study reporting continuous data, both showing no difference between groups (*P*>0.05) [4], [10]. Seven studies remarked about the consistent outcome that two groups had an equal hemostasis effect [6], [9], [21], [25], [26], [27], [28]. The studies concerning septalplasty all mentioned that no septal hematoma was seen during the follow-up period [7], [21], [27], [29].
2. **Bleeding at removal.** Six trials reported bleeding with packing removal. All used similar scales, in which the score indicated bleeding severity. Three studies [25], [27], [29] presented ordinal data, with a cut-point between mild bleeding and moderate/severe bleeding. We then transformed the ordinal data into dichotomous data (see Section 9.2.4, *The Cochrane Handbook* [18]). Meta-analysis showed that the biodegradable nasal packings had a significant protective effect on bleeding caused by packing removal, compared to conventional packings (RR = 0.05; 95% CI = 0.01–0.20; *P*<0.01) (Fig. 3). The I^2^ was 0%, which suggested no heterogeneity. The other three studies provided continuous data [4], [6], [28]. Pooled data also showed that biodegradable packings significantly reduced bleeding at packing removal (SMD = −1.11; 95% CI = −2.18–−0.04; *P* = 0.04) (Fig. 4). However, as this result had high heterogeneity (I^2^ = 95%), we carried out a subgroup analysis. The main heterogeneity source was from the study by Shoman *et al*., in which debridement was performed following the nasal packings removal, causing excess bleeding and pain. The subgroup of “packing removal without debridement” showed more protective effects on bleeding (SMD = −1.69; 95% CI = −1.95–−1.43; *P*<0.01), while the Shoman *et al.* study presented no difference (Fig. 4).

The large sample size study by Vaiman *et al*. was not included in the meta-analysis concerning bleeding at removal [21], which compared fibrin glue (Qiuxi) with Merocel. It only presented the data of bleeding at removal in the Merocel group (43/262 patients developed scanty bleeding and 8/262 developed serious bleeding at removal), whereas the data in the fibrin glue group were not available, as they did not remove the glue.

**Figure 5:**
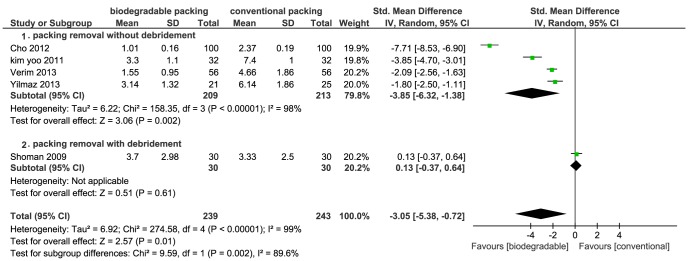
Forest plot of comparison: pain at removal (biodegradable packings versus conventional packings).

**Figure 6:**
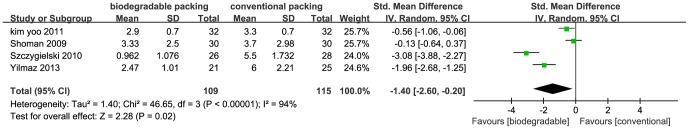
Forest plot of comparison: pain in situ (biodegradable packings versus conventional packings).

**Figure 7:**
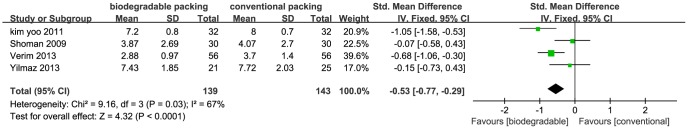
Forest plot of comparison: nasal blockage (biodegradable packings versus conventional packings).

3. **Pain at removal.** There were six studies that recorded pain at packing removal. Four utilized similar Visual Analogue Scales (VAS) with different score ranges [6], [27], [28], [29] and one used a questionnaire scoring system [4]. From pooled data, we found a significant decline of pain in favor of the experiment group (SMD = −3.05; 95% CI = −5.38–−0.72; *P* = 0.01) (Fig. 5). Significant heterogeneity was seen in this result (I^2^ = 99%). However, we could favorably conclude the benefits of biodegradable packings, as each study separately proved a strong decrease in pain at removal in the experimental group, except the study by Shoman *et al.* (for the same previously mentioned reasons). Another study by Kim *et al*. presented dichotomous data [25], proving a remarkably protective effect on pain in the experimental group (RR = 0.05; 95% CI = 0.01–0.37; *P* = 0.003).

4. **Pain in situ.** Four studies recorded pain with *in situ* packing. Three utilized similar VASs with different score range [10], [27], [29]; one used a questionnaire scoring system [4]. Pooled results significantly favored the experiment group (SMD = −1.40; 95% CI = −2.60–−0.20; *P* = 0.02) (Fig. 6). Significant heterogeneity was present in this result, so it must be interpreted cautiously (I^2^ = 99%). During the sensitivity analyses, we removed the study by Szczygielski *et al.* and Yilmaz *et al.* in turn, and found the *P* values changed to 0.08 and 0.10, respectively. This indicated that the pooled result was strongly influenced by these two studies. Nevertheless, the protective effect trend on pain *in situ* was detected in each study.

5. **Nasal blockage** ***in situ*** **.** Four studies investigated nasal blockage with *in situ* packing, using VASs or a questionnaire as mentioned previously [4], [27], [28], [29]. They revealed that biodegradable packings could reduce nasal obstruction as compared to their conventional counterparts (SMD = −0.50; 95% CI = −0.93–−0.07; *P* = 0.02), along with moderate heterogeneity (I^2^ = 67%) (Fig. 7).

- Recovery of nasal mucosa

Postoperative mucosal healing was investigated through endoscopy over several time points during the follow-up period. However, due to the lack of standardized evaluating tools, the methodology which evaluated mucosal healing varied among the studies.

6. **Synechiae/adhesions.** No meta-analysis could be performed due to the differences in methodology and evaluating time within studies. Berlucchi *et al.* recorded a significant difference between the two groups. In particular, at 12 weeks, only 4.6% of MeroGel cases had adhesions as compared to 29.7% in the Merocel patient group (*P*<0.001) [9]. Miller *et al*., in comparing MeroGel with Merocel, used a scale to assess synechiae. They detected no significant difference at two, six, and eight weeks postoperatively, except at four weeks (*P* = 0.049). However, the authors believed it to be a false-positive result [30]. Szczygielski *et al*. evaluated synechiae in eight weeks follow-up, and discovered that synechiae formation occurrence was relatively lower in the CMC packing group, although the difference between the groups was not statistically significant (*P* = 0.092).
7. **Mucosal edema.** Two studies provided detailed information related to mucosal edema. Different types of data made data pooling impossible. Miller *et al.* assessed edema at two, four, six and eight weeks postoperatively, with no difference seen between two groups [30]. Berlucchi *et al*. evaluated edema at two, four, and twelve weeks, only to find MeroGel group had a higher percentage of trophic/normal mucosal appearance at two weeks (11.9%) compared to controls (8.9%), without statistical significance (*P*>0.05) [9].
8. **Infection.** Miller *et al*. evaluated postoperative infection. They found no difference between the two groups (*P*>0.05) [30].
9. **Endoscopic scoring systems evaluating overall mucosa healing.** There were studies that evaluated mucosal healing using different endoscopic scoring systems; these were either validated or not validated. The validated Lund-Kennedy endoscopic scoring system was applied by Cho *et al.* (evaluated at one, two, four, eight, and twelve weeks) and Verim *et al.* (evaluated long-term outcome at one year), both found no difference between groups [6], [28]. Franklin *et al.* utilized a “total endoscopic severity score” (with no detailed information presented) which evaluated mucosal healing. They found a trend toward improvement at 0.5, one, three, and six months in the absorbable (non-significant) group as compared with the non-absorbable group (*P*>0.05) [26].

### 2. Biodegradable packings vs. no packing

- Symptoms associated with packing

In this comparison, it was not necessary to discuss either bleeding or pain at removal, as the relevant studies did not remove the biodegradable dressing.

1. **Postoperative bleeding.** Hemostatic effect related to packing was one of the most significant issues. However, data could not be pooled because of the diverse types of data, evaluation time, and evaluating methodologies.

Antisdel *et al*. used VAS to assess post-ESS bleeding for each group at postoperative day (POD) 1, and found a highly significant reduction in the MPH packed group (*P*<0.01) [22]. In addition, no significant difference was found in POD 7, 17 and 30 (*P*>0.05).

Hu *et al*. reported postoperative bleeding in favor of biodegradable packing (Meropack) [23]. They enrolled 60 children (120 nasal cavities). There were 29 sinuses that underwent lateral wall resection of the conchae bullosa (15 packed, 14 unpacked); four of the unpacked sinuses developed postoperative bleeding. The authors performed a bleeding rate comparison within the two groups in these 29 sinuses, drawing a conclusion that Meropack could significantly reduce bleeding. Though biodegradable packings may reduce bleeding risk in some particular surgical processes, we believe this conclusion is not suitable for all populations undergoing different ESS types.

Kastl *et al.* assessed postoperative bleeding and presented ordinal data, quantified from one day, two weeks, and four weeks postoperatively [31]. No significant differences were found, although the total bleeding incidence (including those with bloody secretions) was slightly higher for the non-packed group than the CMC packed group (*P*>0.05).

Wee *et al.* used a subjective scoring system containing an item related to postoperative bleeding, evaluated at two weeks after FESS. No statistical difference was found from the data. The study recorded three patients out of 21 in the non-packing group had postoperative bleeding which required intervention, while no patients required intervention in the biodegradable packing group (Gelform) [7].

Valentine *et al.* assessed hemostasis at 0, two, four, six, eight, and ten minutes after ESS completion in each group (CD gel on the active side), using Boezaart Surgical Field Grading Scale [14]. They found that the hemostasis time was significantly better for the CD gel side than the control side. A subjective VAS scale assessed postoperative bleeding from day 1 to day 5; no standard deviation was provided. They found no difference between the gel and control groups (*P*>0.05).

**Figure 8:**
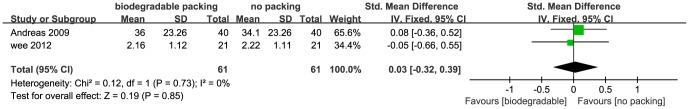
Forest plot of comparison: nasal blockage (biodegradable packings versus no packing).

**Figure 9:**
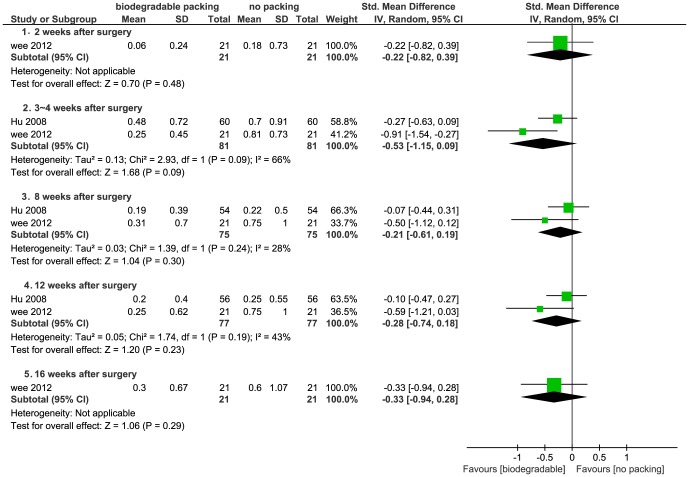
Forest plot of comparison: synechiae (biodegradable packings versus no packing) - for continuous data.

2. **Pain in situ.** This subject was reported from different aspects, thus we did not perform meta-analysis. For example, Andreas *et al.* reported lateralized headache or pressure on the first postoperative day, and Valentine *et al.* reported facial pain/pressure during the first-sixth postoperative days. No significant difference was found between groups (*P*>0.05) [14], [32].
3. **Nasal blockage.** Three studies investigated nasal blockage. Andreas *et al*. evaluated nasal blockage through VAS on the first postoperative day. Wee *et al*. used a subjective symptom score evaluated at one day and two weeks after surgery [7], [32]. We pooled data from the first postoperative day, and found no difference between the packed and unpacked groups (SMD = 0.03; 95% CI = −0.32–0.39; *P* = 0.85) (Fig. 8). Nasal blockage at two weeks in the study of Wee *et al.* was not different between groups (*P*>0.05), either. Valentine *et al*. evaluated nasal obstruction on the first-sixth postoperative days; no standard deviation was provided. They found no difference between groups as well (*P*>0.05).

- Recovery of nasal mucosa

4. **Synechiae/adhesions.** Six studies discussed synechiae assessed through nasal endoscopy. Two of the studies provided continuous data: Hu *et al*. evaluated synechiae at three, eight, and twelve weeks postoperatively and Wee *et al*. evaluated synechiae at two, four, eight, twelve, and sixteen weeks [7], [23]. Data were pooled within subgroups related to different evaluation times [2 weeks (w), 3–4 w, 8 w, 12 w and 16 w]. No differences were detected in this analysis (*P*>0.05) (Fig. 9).

**Figure 10:**
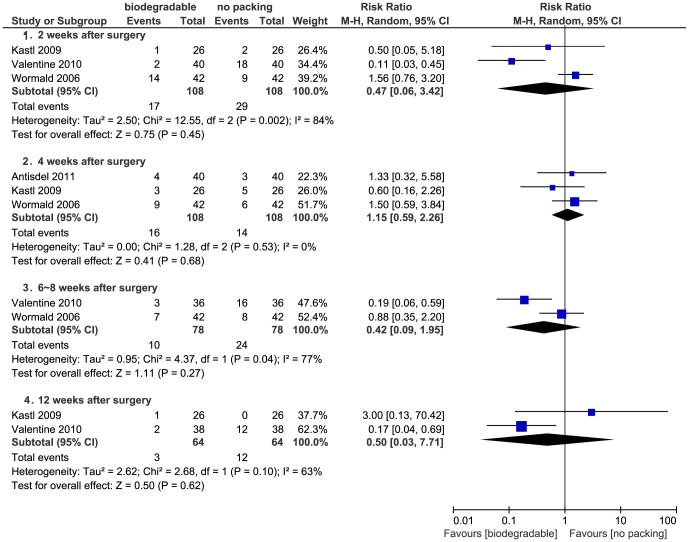
Forest plot of comparison: synechiae (biodegradable packings versus no packing) - for dichotomous data.

Three studies presented dichotomous data. Kastl *et al*. evaluated synechiae at one, four, and twelve weeks, Wormald *et al*. at two, four, and eight weeks and Valentine *et al*. at two, six, and twelve weeks after surgery [14], [33], [34]. Antisdel *et al*. reported synechiae at four weeks in ordinal data [12], which were dichotomized with a cut-point between no synechiae and synechiae formation. Valentine *et al*. reported a protective effect in biodegradable group, while the rest did not. The pooled data in four subgroups (1–2 weeks, 4 weeks, 6–8 weeks and 12–16 weeks) showed no significant differences between groups (*P*>0.05) (Fig. 10).

**Figure 11:**
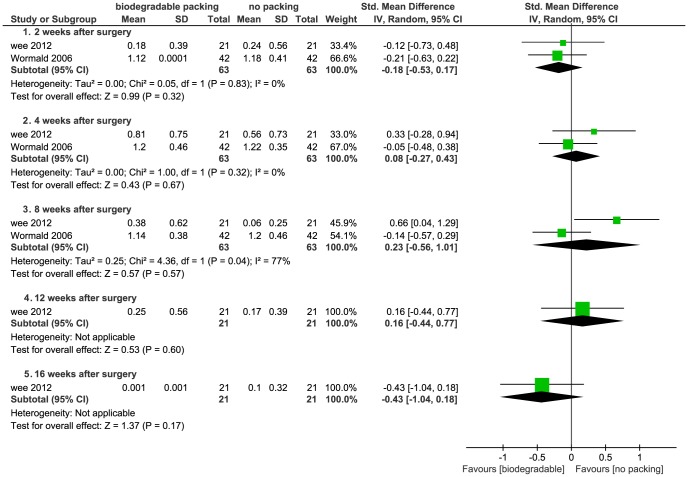
Forest plot of comparison: mucosal edema (biodegradable packings versus no packing) - for continuous data.

**Figure 12:**
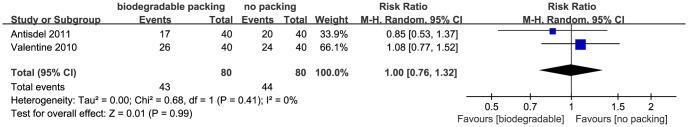
Forest plot of comparison: mucosal edema (biodegradable packings versus no packing) - for dichotomous data.

**Figure 13:**
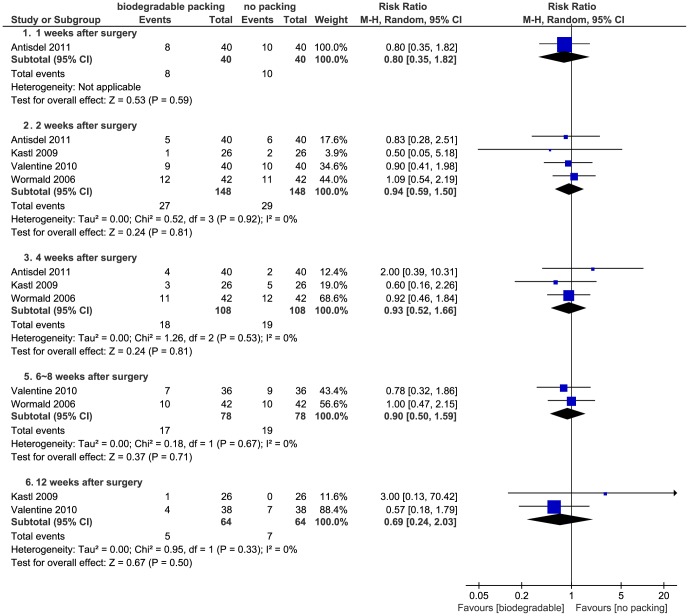
Forest plot of comparison: infection (biodegradable packings versus no packing).

**Figure 14:**
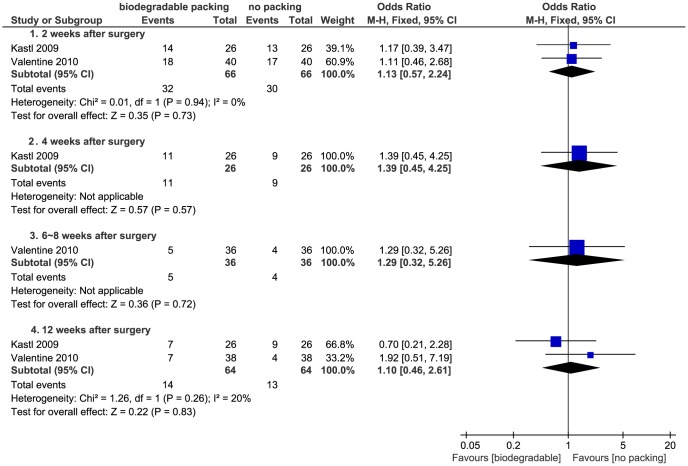
Forest plot of comparison: granulation (biodegradable packings versus no packing).

5. **Mucosal edema.** Wee *et al*. evaluated mucosal edema postoperatively under nasal endoscopy at two, four, eight, twelve, and sixteen weeks. Wormald *et al*. evaluated mucosal edema postoperatively at two, four, and eight weeks [7], [34]. Data were pooled within subgroups of different evaluation times. When a mean or SD = 0, it was imputed by 0.001 to make calculation of the SMD possible, as recommended by *The Cochrane Handbook* [18]. The results indicated that the mucosal edema was statistically equivalent in both the biodegradable packings and non-packing groups (Fig. 11). Two studies presented ordinal data: Antisdel *et al*. reported edema at one, two, and four weeks [12], and Valentine *et al*. at two, six, and twelve weeks [14]. Both studies found no differences between the active and control groups (*P*>0.05). We dichotomized the data which were reported at two weeks; the pooled data showed no significant differences (*P*>0.05) (Fig. 12).

6. **Infection.** Four studies presenting dichotomous data concerning postoperative infection (i.e. ordinal data by Valentine *et al*. and Antisdel *et al*. were dichotomized to correlate with the other studies) [12], [14], [33], [34]; they were evaluated at several time points. Data were processed in the same way in the synechiae, revealing that the infection rate was equal in the two groups (Fig. 13). Another study with continuous data by Hu *et al*. also detected an equal result (*P*>0.05) [23].

7. **Granulation.** Four articles reported post-surgery granulation formation, which was evaluated from week 2 to week 16, postoperatively. The granulation formation rate differed significantly in two articles by Hu *et al.* and Wee *et al*. [7], [23]. For instance, in the non-packing group, the mean score ranged from 0.06±0.23 to 0.68±0.91 in the Hu *et al.* study. The study from Wee *et al.* showed a consistent mean score of zero. We judged that there was a disparity in recognizing “granulation” in the two studies. Thus, meta-analysis was not appropriate. However, they both concluded that there was no difference in granulation between groups (*P*>0.05). Studies by Kastl *et al*. and Valentine *et al.* presenting dichotomous data (or dichotomized ordinal data) indicated the same result [14], [33] (Fig. 14).

## Discussion

Conventional nasal packings refer to a set of common-used removable materials, for instance, ribbon gauze and Merocel. Sometimes they are modified by coating them with chemicals (i.e., iodoform gauze) or glove fingers. Although these modifications can partly improve postoperative outcomes, for example, gloved Merocel has been found to ameliorate the pain at packing removal [36], [37], the innate property of removable packing remain unchanged.

The innovations in nasal packing were motivated by the innate defects of conventional packings in quality of life during early postoperative period. Also, they were expected to improve mucosal healing. Many types of biodegradable materials have been used as nasal packings. Both human and animal trials contributed significantly to the product understanding and their role in ESS [35]. Biodegradable packings either facilitate water uptake (e.g., NasoPore) or contain a substrate to stimulate clotting (e.g., Floseal) to ensure sufficient hemostasis. They are small-sized, located only in the wound area (usually the middle nasal meatus) to provide additional breathing space in the common meatus. They can dissolve within a couple of days before being absorbed by the nose, washed away by nasal irrigation, or removed by suction. Another opinion is not to use nasal packing after surgery.

This systematic review evaluated the current evidence about biodegradable material effectiveness used as nasal packings. We rigidly enrolled RCTs as evidence and then pooled data for meta-analysis where possible.

The results have to be interpreted with caution because there was significant heterogeneity within studies and several results were not robust due to lack of data. The heterogeneity was caused by variety of packing materials, non-standardized surgical process, and methodological differences within studies. As we tested the overall effects of all forms of biodegradable/conventional packings, it's not appropriate to judge the benefit of any specific material over another.

When biodegradable packings were compared with conventional packings, meta-analysis showed significant improvements in postoperative symptoms such as bleeding at removal, pain at removal, pain *in situ*, and nasal blockage *in situ*. The only study that reported contradictory results about symptom related to packing removal was Shoman *et al*., who performed debridement along with removal of both packings. When biodegradable packings were compared with no packing, the studies showed no difference between groups in the symptoms of pain *in situ* and nasal blockage, but the data could not be pooled.

Postoperative bleeding is the key symptom that needs post-ESS observation. Not many disputes have existed related to the hemostatic capability of different packings. The enrolled studies were unanimous in this issue. There are increasing voices in recent years doubting the need for extra packing for ESS and septoplasty in order to prevent bleeding [38], [39]. In the present RCTs comparing biodegradable packings to no packing, two trials favored using biodegradable packings, while three trials showed a comparable bleeding incidence. We speculated that the discordance was partly caused by differences in study components, such as the variety of patient conditions, surgical procedures, and biomaterial types. FESS is not a standardized surgical procedure; its extent and specific surgery nature are determined by the inflammations and anatomical features of the respective sinuses. We noticed that most available literature did not present detailed procedures of ESS for each patient. Save for the study by Hu *et al*. They found Meropack could significantly reduce bleeding, but only in patients who underwent lateral wall resection of the concha bullosa [23]. We cannot yet draw any conclusion about the hemostatic issue of using biomaterial packings or no packing method. More clinical trials are needed with a broader inclusion criteria and more specific groupings.

The non-packing method has some advantages such as decreased sinonasal discomfort, less packing-associated postoperative complications, and less cost. Although significant post-ESS hemorrhage may be rare, some degree of epistaxis can be postoperatively encountered. For example, Kastl *et al.*, Wee *et al*., Antisdel *et al.* and Hu *et al.* reported higher rates of postoperative bleeding (both significant and nonsignificant) in the non-packing group than in the packing group. The “nuisance bleeding” experienced for a few days post-surgery may cause significant anxiety and may negatively impact overall recovery procedure [40]. Anyway, in our opinion, the concept of using no packing is worth further promoting in a prudent manner. This method should be ensured by sufficient intraoperative hemostasis, and close postoperative observation. So far, it may be worthwhile to use biodegradable packing in patients with high postoperative bleeding risk.

The limiting evidence available suggests that biodegradable packings offer patients a better quality of life during the early recovery period than conventional packings. When compared with non-packing group, the data could not be pooled, but all the studies showed comparable morbidities of postoperative symptoms.

As for mucosal healing, synechiae formation is one of the most common post-ESS complications; it can result in recurrent symptoms and subsequent surgical failure. The synechiae usually occur between the middle turbinate and lateral nasal walls. It was expected that degradable packings would avoid synechiae formation, as they could separate two mucosal surfaces in the middle meatus, as well as alleviate the early wound-healing process interruption [26].

However, existing evidence is presently conflicting. Some studies favored biodegradable packings, while the others reported an equal effect to conventional packings/no packing. Some of these conflicting results may be attributed to various surgical techniques, as well as different postoperative management regimens. Besides, mucosal healing may vary with different biomaterials. Biological products such as fibrin, collagen, or thrombin based materials will be potentially different than non-biologic CMC, Nasopore, etc. None of the materials used in the enrolled RCTs were reported to have a significant detrimental effect on synechiae formation. Nevertheless, other research not included in this review reported important shortcomings. Tom *et al.* used gelatin film for pediatric patients who underwent FESS. They found gelatin film increased synechiae formation, as compared with the non-packing group [41]. One double-blind randomized controlled trial showed both increased adhesion formations and granulations, as compared to Floseal (a paste of bovine gelatin particles combined with thrombin) with thrombin-soaked gelatin foam [42]. This was confirmed by a large retrospective case series [43]. A rabbit model showed that Floseal increased fibrosis and was incorporated within the healing mucosa [44]. Explanation for this effect may be as a result of the bidirectional relationship between coagulation and inflammation; strong initiation of the coagulation cascade resulted in strong activation of inflammation and fibrosis pathways [45]. Thus, these materials should be cautiously used as nasal packing for patients with a high risk of synechiae formation.

Postoperative mucosal edema, infection, and granulation formation are other aspects associated with mucosal healing. Our study revealed that there was no difference between biodegradable packings and conventional packings or non-packing groups. Hence, it is not appropriate to assert that biodegradable packings can reduce synechiae formation and improve mucosal healing.

Conventional packings may cause complications such as septal perforation, aspiration, toxic shock syndrome, foreign body granuloma, obstructive sleep apnea (secondary to nasal obstruction), and even death [46], [47]. Biodegradable packing complications are dependent on their materials. A murine model study by Jacob *et al.* suggested MeroGel (made of hyaluronic acid) may have osteogenic potential [48]. Allergic or neurotoxic reactions to one of the Quixil constituents may occur [11]. Collagen products (porcine source) including gelatin films may increase synechiae and granulation tissue formation [41]. Animal product derivative substances can carry serious risks of antibody formation and potential disease transmission [12]. Bovine thrombin preparations (Floseal) may induce coagulation factor antibodies, developing serious bleeding complications [49]. However, RCTs included in this review did not encounter these complications.

Several limitations related to our meta-analysis should be considered. First, all included RCTs in our study were published in English. In addition, considerable heterogeneity was seen in some outcomes, and there was insufficient information presented in several outcomes to get robust results. At last, as we gathered all forms of degradable packings in one group as well as all forms of conventional packings in another group, it is impossible to determine the effect of any specific packing over another.

Rhinologists have been trying to improve postoperative care in patients undergoing endoscopic sinonasal surgeries, thereby giving rise to a thriving nasal packing industry. Abundant products have entered the market; however, clinical trials for each specific product remain elusive. In this review, we evaluated the overall effects of biodegradable packings for comparison with the conventional packings or non-packing groups. It may help to understand the current status of different packing methods, but we recommend caution when putting any specific one into use.

## Conclusions

Our meta-analysis suggested biodegradable nasal packings can offer patients a good quality of life during the early recovery period. This would be significantly better than conventional packings and probably comparable to the non-packing group. However, no beneficial or detrimental effects on postoperative mucosal healing were established based on existing evidence. In situations where the surgeon feels post-operative packing is necessary, we would recommend and the evidence would support the use of biodegradable packing in lieu of conventional packing. This analysis did not statistically favor the use of bio-degradable over no-packing at all. However, we would recommend its use when the risk of post-operative bleeding is high, despite no definitive evidence to support this. We advocate more large scale, multi-center and well-designed RCTs to testify for both the effectiveness and safety of nasal packing materials.

## Supporting Information

S1 Checklist **PRISMA 2009 Checklist.**
(DOC)
